# Research on the Electrosensitivity and Electrothermal Properties of Intelligent High-Performance Concrete Materials

**DOI:** 10.3390/ma17010054

**Published:** 2023-12-22

**Authors:** Yunlong Zhang, Huichao Sun, Xuesong Qian, Jing Wang, Guojin Tan

**Affiliations:** 1 Key Laboratory for Comprehensive Energy Saving of Cold Regions Architecture of Ministry of Education, Jilin Jianzhu University, Changchun 130118, China; zhangyunlong@jlju.edu.cn; 2 College of Traffic Science and Engineering, Jilin Jianzhu University, Changchun 130119, China; sunhuichao@student.jlju.edu.cn (H.S.); wj242827@jlju.edu.cn (J.W.); 3 College of Transportation, Jilin University, Changchun 130025, China; tgj@jlu.edu.cn

**Keywords:** high-performance concrete, carbon fiber, basalt fiber, resistivity, self-sensing property, electrothermal property

## Abstract

In order to enhance traditional building materials, High-performance concrete (HPC) is being modified by adding carbon and basalt fibers with volume contents of 0.75–1.25% and 0.15–0.35%, respectively. The original mechanical properties are maintained while developing the material’s intelligent self-sensing and self-heating functions, which are tested for pressure sensitivity and bending sensitivity, and with electrothermal tests. The results demonstrate that carbon fiber can significantly reduce the matrix resistivity of high-performance concrete, reaching the percolation threshold at a content of 1%. The inclusion of basalt fibers in the material results in a decrease in resistivity. However, the addition of mixed fibers leads to improved mechanical–electrical sensitivity under compression and bending, with a positive hybrid effect. The optimal contents for carbon fiber and basalt are 0.75% and 0.3%, respectively. In electrothermal tests, the specimen can reach a temperature of 104.5 °C with a heating rate of 25.86 °C/h, indicating the potential for self-monitoring and the electric melting of ice and snow. These findings provide support for the intelligent improvement of building structures in the new era.

## 1. Introduction

The term ‘intelligence of concrete material’ refers to the ability of a structure to perceive environmental conditions and respond accordingly. This includes the material’s self-sensing and self-heating functions. Researchers have long been dedicated to developing a new type of cement-based material that not only boasts excellent mechanical properties but also has certain intelligent capabilities. This is in order to achieve a comprehensive replacement of traditional concrete materials to maximize the benefits of intelligent functions for building structures. High-performance concrete (HPC), a new type of concrete with high strength and durability, has matured after years of development. It has the potential to overcome the limitations of traditional concrete and serves as an ideal intelligent, improved matrix of building materials [1,2].

The effective intelligent functioning of concrete material primarily relies on the conductive properties of its matrix. HPC is typically produced using a lower water–binder ratio and a higher quantity of cementitious materials [3]. The conductive ability of a capillary solution is similar to that of a semiconductor, and its volume resistivity is usually within the range of 103 Ω·cm to 108 Ω·cm. This ability mainly depends on the directional movement of positive (K^+^, Na^+^, Ca^2+^) and negative (OH^−^) ions in the solution. When the solution is wet, the resistivity can reach 103 Ω·cm to 104 Ω·cm. The conductive stability of a cement matrix is influenced by various factors, such as the cement type, moisture content, hydration degree, and environmental conditions. However, the conductive ability is susceptible to external factors, making it challenging to achieve consistent intelligent performance in traditional settings. Therefore, there is an urgent need for improvement in this area [4,5,6,7].

Research has demonstrated [8,9,10] that incorporating conductive fillers into concrete materials can significantly enhance their resistivity. Moreover, this addition can induce a piezoresistive effect when subjected to a load, meaning that the changes in resistivity accurately represent the internal stress–strain variations within the structure. To address the issue of significant changes in resistivity in cement-based materials, it is necessary to incorporate various types and amounts of conductive fillers. This will help to decrease the reliance of cement-based materials on ionic conductivity within capillaries, ultimately leading to the attainment of stable electrical conductivity.

The addition of conductive fillers to HPC not only effectively improves the piezoresistive rate but also increases the matrix’s electrical–thermal conversion ability. This is due to the Joule effect, which is the continuous heat release produced by the material under the action of an external power supply [11,12]. This characteristic can be used to achieve the functions of self-deicing and melting snow on road surfaces in winter in seasonally frozen areas. Compared to traditional snow removal methods, this approach has a relatively low cost, simple operation, causes less structural damage, and has a wide range of potential applications [13,14,15,16]. 

Salam R Armoosh [17] conducted a study on enhancing the electrical conductivity of cement composites using metal materials. The self-heating properties of these composites were tested to examine their resistance heating capabilities. The aim was to assess the feasibility of utilizing metal reinforced cement composites as affordable heating elements for large-scale heating purposes. Farzana [18] proposed an innovative approach for sustainable and environmentally friendly road maintenance in winter. The design involves an electrothermal concrete panel incorporating carbon fiber and carbon nanotubes as dielectrics. Through careful optimization of the mix ratio and geometric layout, the concrete panel achieves efficient electrical heating. When the power density ranges from 1200 to 1800 W/m^2^, the surface temperature can rise by up to 20 °C within 2 h.

After years of development, the variety of conductive fillers has increased. Currently, carbon fiber conductive materials are the main fillers due to their excellent performance-to-price ratio [19,20,21]. Carbon fiber is an excellent reinforcing concrete fiber with high tensile strength, stable chemical properties, and good electrical conductivity. It can effectively enhance toughness, delay cracking, and significantly reduce the resistivity of the matrix, resulting in the functionalization and upgrading of the material [8,22]. Bontea [23] investigated the ability of carbon fiber cement-based materials to detect damage under dynamic load. The study found that the elastic modulus of the matrix decreases and the resistivity changes irreversibly when high levels of damage occur. Chen [24] further explored the self-sensing performance of carbon fiber cement mortar sensors at different notch depths and confirmed the possibility of crack monitoring. The testing process showed that the maximum crack width can reach 0.275 mm. Ayoub Dehghan [25] conducted a study on the piezoresistivity of hybrid ECCs. These ECCs were developed by combining polyvinyl alcohol with shape memory alloy fibers, steel fibers, and carbon fibers individually. Among the composites containing carbon fibers, the hybrid ECC with 0.6% CFs demonstrated the most favorable piezoresistive behavior when subjected to compressive stress cycles. This was evident from its high correlation coefficient between the fractional change in resistivity and the strain, a narrower 98% prediction interval, and fewer noisy electrical signals.

However, the potential improvements in the mechanical properties of materials by using carbon fiber are limited; therefore, the cement needs to be further strengthened by using additional fibers. Basalt fiber has gained popularity in recent years due to its exceptional tensile strength and expansion properties. The natural silicate composition of basalt fiber allows for a seamless integration with concrete [26,27]. Zielinskin and Olszewsk [28] discovered that the use of basalt fiber in concrete has an impact on its compression, bending, and plastic shrinkage, and significantly affects its strength and fluidity. Furthermore, the addition of basalt fiber has been found to effectively enhance the tensile strength and ultimate elongation of concrete when compared to ordinary concrete. It is believed that the matrix can maintain its connection state in the failure state, preventing explosion collapse [29,30]. However, at present, there are few studies on the properties of the two kinds of fibers, which need to be investigated in depth.

This study investigates the use of mixed carbon fiber and basalt fiber as reinforcing materials in HPC to explore the intelligent function of materials under different fiber contents. The research resulted in the development of a new functional HPC that can address the high costs and poor adaptability issues of traditional monitoring sensors. The new concrete can also effectively improve road traffic conditions after snow, making it significant to the research and popularization of new intelligent structures in seasonally frozen areas [31,32,33].

Currently, there is a lot of research being conducted on upgrading and improving traditional building materials. After further improvements in their function, these materials will be used in various construction projects instead of regular concrete, thus becoming the true ‘21st century concrete’.

## 2. Materials and Methods

### 2.1. Materials

HPC typically comprises a combination of cement, cementitious materials, fine aggregates, synthetic fibers, admixtures, and water.

The material was P.II 52.5 cement produced by the Jilin Yatai Cement Industry (Changchun, JilinProvince, China). The cement strength and quality indices met the General Portland Cement Testing Standard (GB175-2007) [34]. The silica fume and grade I fly ash was produced by the Shengyun Mineral Products Factory in Lingshou County and the S95-grade ore powder, the properties of which are shown in Table 1, was produced by Xibaipo New Energy Co., Ltd., Xibaipo, Hebei Province, China. The quality of the cementitious materials conformed to the Technical Specification for the Application of Mineral Admixtures (GB/T51003/2014) [35]. The manufactured sand produced by the Changchun Qingfeng Mining Industry was used to replace the quartz sand in traditional HPC, and its fineness modulus was 2.69.

The addition of fiber is a crucial factor in enhancing the performance and intelligent function of materials. In this study, a 6 mm long carbon fiber manufactured by Jiangsu Chuangyu Carbon Fiber Technology Co., Ltd., Yancheng, Jangsu Province, China. was selected. The carbon fiber was polypropylene clear (PAN)-based and its performance index is presented in Table 2. To further reinforce the material, a basalt fiber with a length of 12 mm and a diameter of 17.4 μm, produced by Shanghai Shenqi Chemical Technology Co., Ltd, Shanghai, China. was used as an auxiliary reinforced fiber. The performance index of the basalt fiber is shown in Table 3.

The superplasticizer adopted was a polycarboxylic acid series high-performance superplasticizer produced by Chongqing Tengzhi Science and Technology Co., Ltd, Chongqing, China. which was used to adjust the mixing performance of the mixture.

### 2.2. Specimen Preparation

The uniformity of fiber dispersion during specimen preparation significantly affects the material’s properties. To ensure the fiber was evenly dispersed and effectively enhanced the final material properties, the following methods were employed:

To ensure even mixing, the weighed cement, fly ash, silica fume, mineral powder, and machine-made sand were added into the concrete mixing pot using a single horizontal shaft-forced concrete mixer produced by Beijing Century Chengda Instrument Manufacturing Co., Ltd., Beijing, China.

The dry material continued to be stirred, adding the two kinds of fibers in batches during the stirring process. First, the high-quality carbon fiber was sprinkled through the sieve and stirred for 2 min; then, the accurately weighed basalt fiber was sprinkled and stirred for 2 min to observe the existing state of the fiber and ensure that the fiber was dispersed evenly.

In the process of dry material mixing, the weighed superplasticizer was dissolved in water. The solution of superplasticizer was added to the mixed dry material and stirred for 2~4 min until it was formed.

After mixing, the mixture was poured into the designated test mold and electrodes were inserted into the sample to be tested for electrical properties. The mold was then placed on a vibrating table and vibrated until water was secreted from the surface of the test piece to ensure proper compaction. The test piece was then moved to a designated location and covered with plastic film to prevent moisture evaporation. After 24 h of natural curing, to simulate the construction maintenance environment in cold areas during autumn and winter, the test’s curing temperature was maintained at 13 ± 2 °C. Additionally, the film was covered with water every 8 h after the final setting to ensure adequate moisture during the curing process.

### 2.3. Test Method

Based on the results of preliminary laboratory tests, the basic mix ratio for maximizing the improvement in the intelligent function of HPC in this study was determined by referring to numerous relevant research papers [36]. Table 4 displays the final mix ratio.

The test fiber was designed according to the volume content, considering that the two kinds of fibers were non-metallic fibers. Too high a content will make it more difficult to stir and prone to agglomeration; so, in addition to the control plain HPC, the total fiber volume should be limited to between 0.75% and 1.25% of the matrix volume, in addition to controlling plain HPC. To maximize the intelligent function of HPC, the test group stipulated that the content of carbon fiber should not be less than 0.75%. This ensures that the material consistently maintains a lower resistivity. For more details on the specific test fiber contents, please refer to Table 5.

#### 2.3.1. Resistivity Measurement

In this experiment, a copper net was chosen as the material for connecting electrodes, and resistivity was measured using the DC two-electrode method and the volt–ampere method. Prior to testing, a DC voltage of 2.0 V was applied to the pre-embedded electrodes at both ends. Due to the polarization effect in the matrix after electrification, the current was unstable. Therefore, a loading test was carried out after the current balance stabilized by connecting the specimens in series and recording the current value after it became stable. The resistivity changes in the specimens at different ages were calculated according to Ohm’s law. In order to reduce the error, the average resistivities measured by 100 mm × 100 mm × 100 mm, 300 mm × 100 mm × 100 mm and 400 mm × 100 mm × 100 mm were compared.

#### 2.3.2. Pressure Sensitivity Test

**Figure 1:**
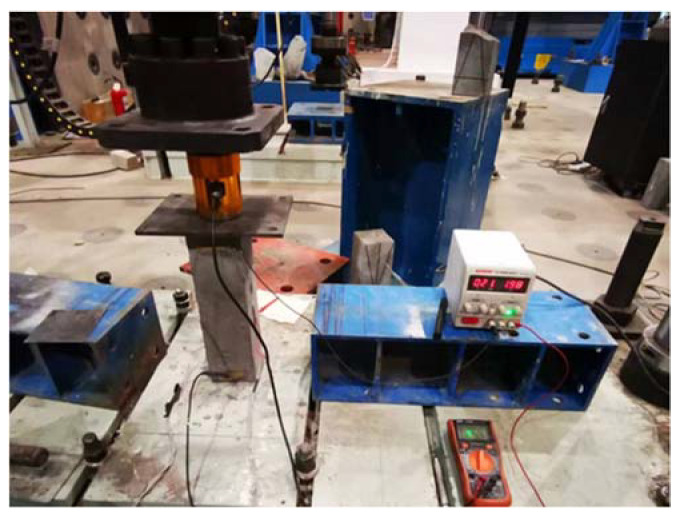
Pressure sensitivity test method.

The pressure sensitivity test is commonly employed to assess the correlation between the alteration in material resistivity and the variations in pressure and compressive strain resulting from a uniform change in compressive load. The circuit was connected in the same mode as the resistivity test. Strain gauges on both sides of the specimen were connected to the strain collection box. The specimen was then placed on the press for cyclic load loading, with current and strain changes recorded every 5 s during measurement. In order to ensure that the test was always conducted within the elastic range of the material, the maximum loading load of the specimen was controlled to be within 1/3 of the material’s strength. Additionally, to prevent tipping of the specimen, the lowest load of the test was set at 5 kN, the size of the specimen was 300 mm × 100 mm × 100 mm, and the test process is shown in Figure 1.

#### 2.3.3. Bending Sensitivity Test

**Figure 2:**
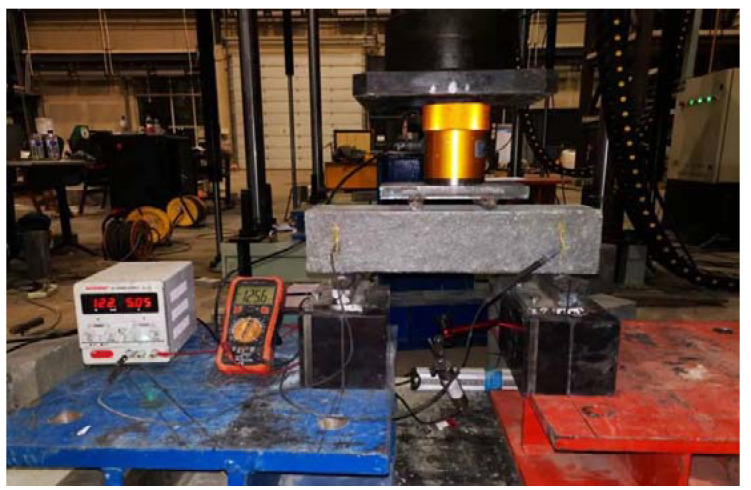
Bending sensitivity test method.

This study aimed to evaluate the self-sensing ability of intelligent HPC under repeated bending load. To achieve this, a specimen with dimensions of 400 mm × 100 mm × 100 mm was used, and a reserved electrode connection circuit was employed during pouring. Four-point bending was applied at a loading rate of 0.15 mm/min, and the change in current was recorded every 5 s until the specimen’s bearing capacity was lost and fracture occurred. The change in resistivity was analyzed based on the load and deflection. The bending sensitivity of the material was evaluated, and the test process is shown in Figure 2.

#### 2.3.4. Electrothermal Test

**Figure 3:**
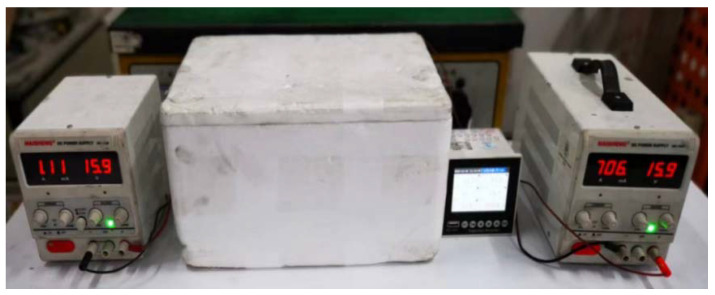
Electric heating test method.

To investigate the temperature rise of the material, the circuit was connected in series following Joule’s law. A 16 V DC voltage was applied to a 100 mm × 100 mm × 100 mm concrete specimen for 3.5 h. To minimize heat loss, a 40 mm thick thermal insulation foam board was wrapped around the concrete specimen, and temperature sensors were placed on the top and bottom of the concrete test block. These sensors were connected to a paperless recorder to collect temperature data, which were automatically recorded every 10 s. Figure 3 illustrates that the test was conducted in a general indoor environment during winter.

## 3. Results and Discussion

### 3.1. Resistivity

#### 3.1.1. Effect of Curing Age on the Resistivity of Materials

The curing of concrete is a lengthy process, which continuously changes the structure and composition of the matrix due to the hydration reaction. This change can also affect the resistivity of intelligent HPC. To fully analyze the matrix resistivity changes during the curing process, different groups of specimens were measured at 1 d, 3 d, 7 d, 14 d, 21 d, and 28 d, based on two mainstream conductive mechanisms of cement-based materials: the seepage theory and the tunnel effect.

**Figure 4:**
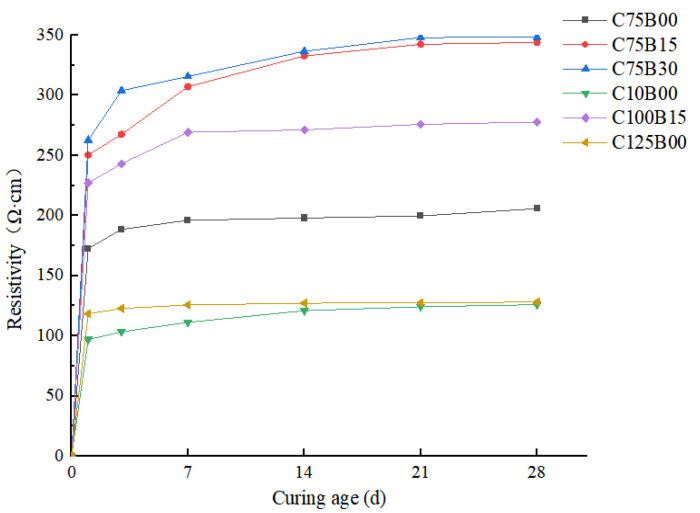
Effect of curing age on resistivity.

The specific resistivity changes with the nursing age are shown In Figure 4.

The test results indicate that the resistivity of the concrete matrix generally increases with the gradual increase in curing age, and this increase can be divided into three stages: rapid growth, slow increase, and stabilization. During the rapid growth stage, which occurs during the first 1 to 7 days of curing, the resistivity of each fiber content increases rapidly, with a maximum growth rate of 22.65%. The slow rising stage occurs between 7 and 21 days, during which the resistivity growth rate slows down, with an average increase of 6.52%. The last stage is observed during curing from 21 to 28 days of molding, during which the resistivity increase is not significant, at less than 4%. This indicates that the material resistance value reached a relatively stable state at this stage.

The resistivity of concrete is affected by the curing age due to changes in the internal material composition and structural state caused by hydration and the pozzolanic reaction in the matrix. Initially, a large amount of free water in the concrete increases the ionic conductivity of the matrix, resulting in relatively low resistivity. However, as the hydration reaction progresses, free water reacts with cementitious materials and gradually converts into bonded and gelled water with poor electrical conductivity. During the formation of hydration products, the internal structure of the material undergoes changes, which result in an increase in the insulation barrier between conductive materials. This gradual increase leads to a decrease in the passing rate of conductive particles, ultimately resulting in a rapid increase in material resistivity during the early stages. As time passes, the rate of hydration slows down, and the free water content and insulation barrier change at a slower rate, resulting in a relative decrease in the resistivity growth rate. During the final stage of curing, the conductive filler constructs a relatively complete conductive network. Although hydration and pozzolanic reactions continue, their effect on the conductive network is greatly reduced. As a result, the resistivity of the matrix changes slowly and tends to stabilize.

#### 3.1.2. Effect of Fiber Content on the Resistivity of Materials

**Figure 5:**
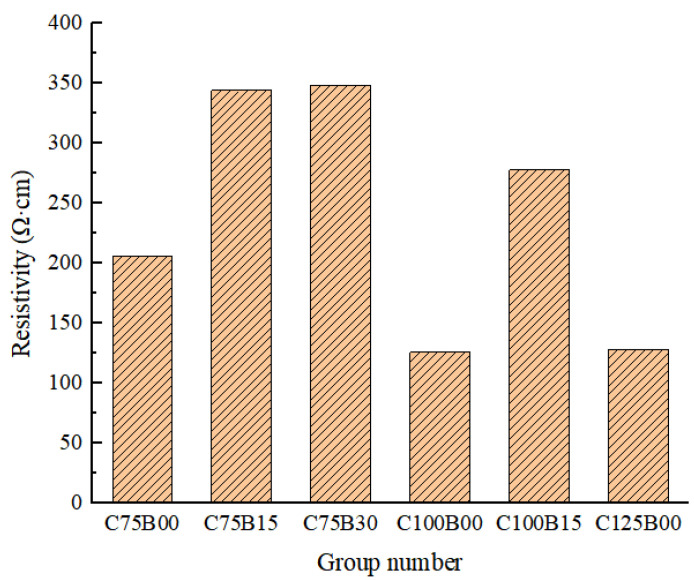
The 28 d resistivity of hybrid fiber HPC.

Figure 5 displays the 28-day resistivity of intelligent HPC, which has been mixed with varying amounts of fiber.

The experimental results show that the 28 d resistivity of the material decreases first and then increases with the increase of carbon fiber content. Compared with plain HPC, the addition of carbon fiber changes the resistivity of the matrix by several orders of magnitude. When the carbon fiber content increased from 0.5% to 0.75%, the material resistivity decreased from 785.47 Ω·cm to 205.86 Ω·cm, showing a huge decrease of 73.79%. When the carbon fiber content continues to increase to 1.0%, the decrease in the resistivity of the material slows down to 38.79%, reaching a minimum resistivity of 126 Ω·cm. When a 1.2% carbon fiber is added, the resistivity increases slowly, reaching 128.14 Ω·cm, showing an increase of 1.7%, and the upward trend is not obvious. However, this also shows that, with the increase in carbon fiber, the construction of the conductive path inside the matrix is gradually complete. In addition, it finally reaches the permeability threshold when the carbon fiber content is 1.0%, and the resistivity decreases to its lowest. At this time, the continuous increase in carbon fiber content will lead to the resistivity increase in the material. 

The study reveals that the addition of basalt fiber leads to an increase in the resistivity of the matrix, resulting in a negative electrical effect regardless of the carbon fiber content. The extent of this effect decreases with an increase in basalt fiber content. The C100B15 group exhibited the best conductive effect among all the HPC mixed with fiber, with a resistivity of 277.86 Ω·cm. This value was 64.63% greater than that of concrete with only 0.5% carbon fiber. The introduction of basalt fiber enhances the matrix strength, but its length–diameter ratio is too large and lacks electrical conductivity, hindering the smooth flow of the conductive network when built with carbon fiber. As a result, HPC mixed with basalt fiber experiences a gradual increase in matrix resistivity with increasing basalt fiber content. However, when the carbon fiber content approaches the percolation threshold, resistivity reaches its lowest value and the side effect of basalt fiber is limited, resulting in reduced effectiveness.

The introduction of basalt fiber into the material results in a slight decrease in its electrical conductivity. To comprehensively evaluate the impact of this hybrid fiber, other electrical properties, such as the material’s mechanical–electrical conversion sensitivity, should be further investigated. This will enable the selection of the optimal ratio of mixed fibers and provide support for the development of intelligent HPC materials.

### 3.2. Pressure Sensitivity

**Figure 6:**
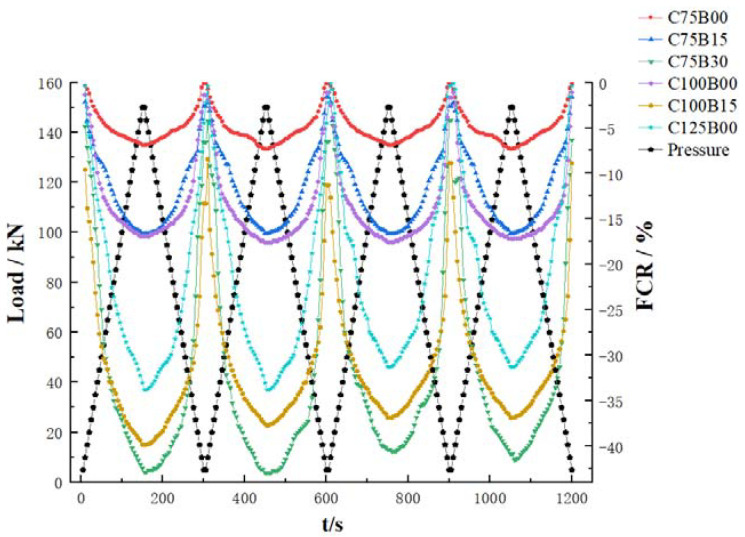
Test results of the pressure sensitivity of HPC with different fiber contents.

The electrical properties of HPC are largely determined by the content of the conductive fiber, and this also affects the varistor properties of the material. To standardize test conditions, external test factors were comprehensively analyzed to determine the pressure sensitivity of each test material with varying fiber contents. A sample size of 100 mm × 100 mm × 300 mm was used, with a cyclic loading rate of 1 kN/s and a maximum load of 150 kN. During the test, ammeter readings were recorded every 5 s, and the material’s resistivity was calculated. Figure 6 shows the specific test results.

To directly describe the variation amplitude of resistance, two indices have been introduced, namely the fractional change in resistivity (*FCR*) and the gauge factor (*GF*):

(1) $$FCR=\frac{\rho -\rho_{0}}{\rho_{0}}\times 100\%$$

(2) $$GF=\frac{FCR}{\Delta \varepsilon }=\frac{\Delta \rho /\rho_{0}}{\varepsilon_{i}-\varepsilon_{0}}$$

In the formula, *ρ* is the resistivity in the matrix during loading of the specimen in Ω·cm; *ρ*_0_ is the initial matrix resistivity of the specimen in Ω·cm; *ε_i_* is the strain of the specimen when tested at any time; and *ε*_0_ is the specimen change at the beginning of the test.

**Figure 7:**
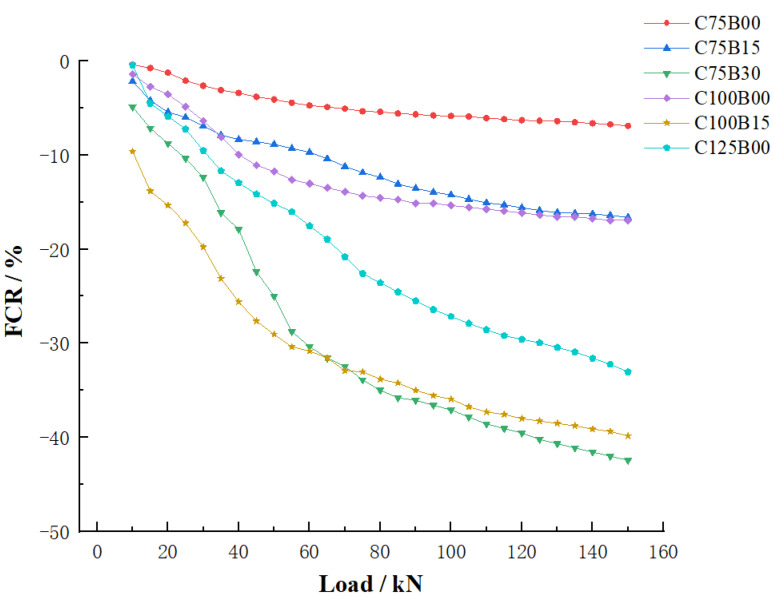
Variation in the compressive resistivity of HPC with different fiber contents.

Figure 7 depicts an analysis of the resistivity change rate of various materials under different levels of pressure. The test results indicate that the HPC material exhibits good pressure-sensitive properties when mixed with either carbon or basalt fiber. Additionally, resistivity displays a consistent upward and downward trend with the cyclic application of load, demonstrating a unified corresponding relationship between the resistivity and the load value of concrete. The distribution of sampling points shows a scattered pattern at low loads but becomes denser as the load value increases. This is attributed to the small pressure and greater distance between conductive fillers, resulting in a significant reduction in material volume under the same pressure. The reduction in fiber spacing has resulted in a more compact lap and a noticeable decrease in resistivity. As the pressure approaches the maximum bearing value within the elastic range, the volume of the material shrinks to near its limit state. This results in dense connections in the conductive fiber network, which cannot be further reduced, limiting the change in resistivity to each individual sample. Additionally, the test results indicate that the amount of mixed fiber has a significant impact on the varistor properties of the material.

**Figure 8:**
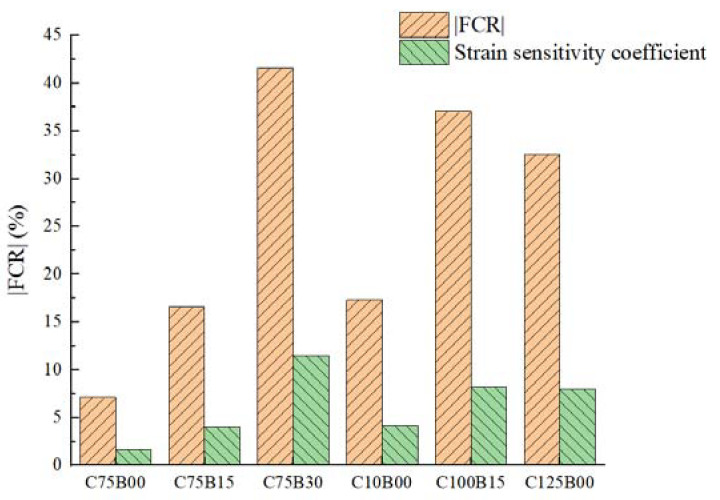
Electrical property parameters of HPC materials with different fiber contents.

In order to investigate the pressure sensitivity of concrete materials with varying fiber contents and the resulting change in material stress during compression, the sensitivity coefficients of the HPC material with different fiber contents were calculated and are presented in Table 6. Additionally, the electrical properties of HPC materials with varying fiber contents are depicted in Figure 8 for comprehensive analysis.

The test results indicate that the strain sensitivity coefficient and resistivity change rate of HPC materials exhibit a consistent overall trend. When only carbon fiber is mixed, both the FCR and GF values increase with an increase in fiber content. However, when mixed with basalt fiber, these values are inconsistent with the resistivity results. The addition of basalt fiber leads to an increase in both FCR and GF values, indicating a reverse mixing effect of fibers. This effect reaches its peak when the carbon and basalt fiber contents are 0.75% and 0.3%, respectively, resulting in the best sensory performance.

Previous studies have indicated that carbon fiber dispersed in HPC relies on the formation of a conductive network to facilitate the transmission of electrical signals and achieve matrix conductivity [19,37,38]. Based on the analysis of the test results, it can be inferred that a lower carbon fiber content leads to a wider distance between conductive networks and a thicker gel medium between fibers. This results in higher insulation barriers and difficulty for conductive particles to pass through. However, when the material undergoes significant deformation, the spacing between carbon fibers decreases, leading to an increase in the passing rate of conductive particles. Furthermore, as the carbon fiber content increases, the conductive path tends to improve, resulting in a higher efficiency of particle transfer. Consequently, the resistance of the material undergoes noticeable changes when deformed with a substantial amount of carbon fiber.

**Figure 9:**
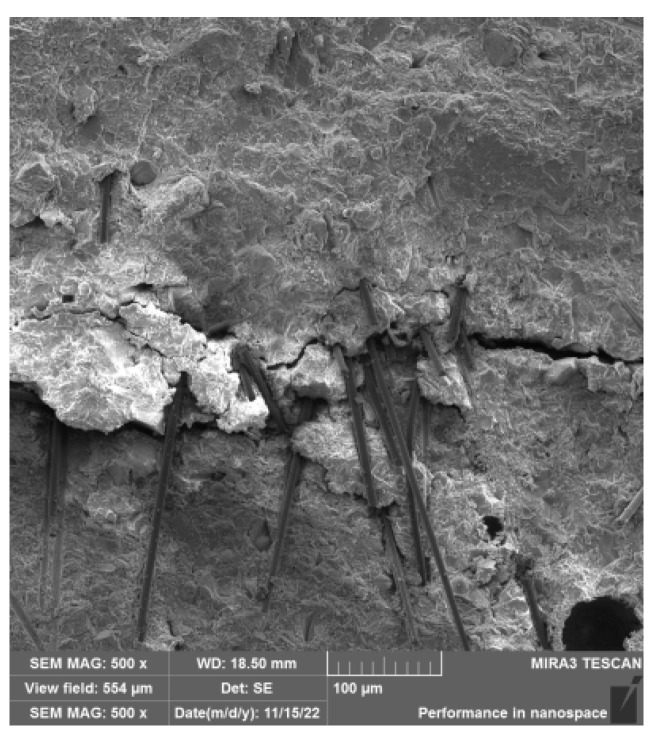
Microstructure diagram of failure morphology of intelligent HPC.

As is depicted in Figure 9, the microstructure analysis of the material reveals that the presence of mixed fibers in the mixed system enhances the integrity of the matrix. This acts as a bridging mechanism, impeding the formation and progression of microcracks, while reducing defects in the matrix and insulation barriers. During concrete production, the two types of fibers are thoroughly mixed and fused, resulting in a complex interweaving and the formation of a comprehensive fiber network. Furthermore, the inclusion of basalt fiber serves as a transmission medium, facilitating a closer relationship between carbon fibers. This leads to a reduction in the extent of cracking in the material matrix and enhances the likelihood of overlap between long-distance carbon fibers during loading, failure, and deformation. Consequently, it ensures the smooth transmission of electrical signals, thereby influencing the change in the resistivity of the matrix. The material exhibits a clear improvement in performance in terms of electrical sensitivity.

The results of the pressure-sensitive performance test indicate that the combination of intelligent HPC material and fiber can effectively demonstrate the loading state during compression. The C75B30 group showed the most significant impact, offering the potential for overall structure monitoring in intelligent HPC structures.

### 3.3. Bending Sensitivity

**Figure 10:**
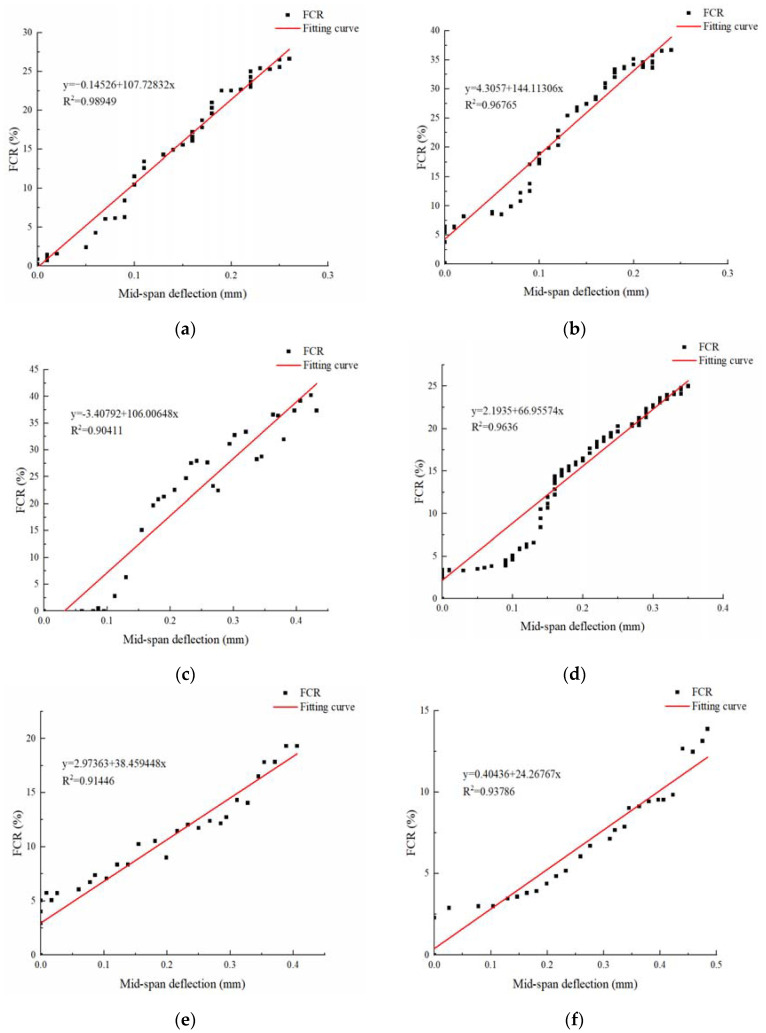
Results of bending sensitivity tests of HPC with different fiber contents. (**a**) C75B00; (**b**) C75B15; (**c**) C75B30; (**d**) C100B00; (**e**) C100B15; (**f**) C125B00.

The results of the flexural strength tests of intelligent HPC are shown in Table 7. As the fiber content increases, the degree of improvement becomes more apparent. However, even with the addition of fibers, the specimen still exhibits a brittle failure mode when subjected to bending. To analyze the stress sensitivity of materials under bending, the deflection–resistivity change rate curve of intelligent HPC with varying fiber contents was plotted using the absolute values of the FCR data. This is depicted in Figure 10.

The test results indicate that each smart HPC material exhibits varying peak resistivity change rates when subjected to bending at different degrees. The change rate for C75B00 reached 26.64%, while C75B15 showed a change rate of 36.67%. The change rate for C75B30 was even higher, reaching 37.35%. Subsequently, the resistivity change rate of the group decreased. Specifically, C100B00 had a change rate of 25.01%, C100B15 showed a change rate of 20.30%, and the change rate for C125B00 was the lowest at 13.88%.

The study demonstrated that HPC with varying fiber contents exhibited favorable bending sensitivity. As the amount of carbon fiber increased, the maximum deflection of the material continued to rise, while the range of resistivity decreased. On the other hand, an increase in basalt fiber resulted in an upward trend in both the maximum deflection and resistivity, and the fibers exhibited a positive hybrid effect.

HPC exhibits high compressive strength. Under bending load, the upper compression zone of the concrete matrix is compressed, resulting in a decrease in the spacing of conductive fillers and a denser lap, leading to a further decrease in resistivity. However, the lower tension area experiences gradual destruction and the presence of microcracks impedes current propagation, resulting in an increase in resistivity. HPC has greater compressive strength than tensile strength, leading to brittle failure when bending. Additionally, the resistivity in the tension area is limited and changes differently to materials with good ductility. During bending, the resistivity decreases continuously until sudden fracturing occurs at the maximum bearing strength, and the resistivity decreases to zero.

In order to achieve real-time self-monitoring of structural compression, the change in resistivity was measured to determine the deformation and damage of components. The parameters selected for this measurement were the resistance change rate and deflection. The relationship between these two parameters was established by fitting a curve based on the test results, resulting in a force–electrical signal relationship. The degree of correlation between deflection and the resistivity change rate during the bending process was analyzed, and the potential for practical engineering implementation is discussed.

A first-order function is used to express the change rate of material resistivity (*y*) and deflection (*x*), with the fitting expression shown below:

(3) y=a+bx

In the formula, *y* is the resistivity change rate (FCR) percentage; *x* is the deflection in mm; *a* is the longitudinal intercept; and *b* is the slope.

The size of *a* and *b* is only related to the type and amount of fiber. The table shows a correlation coefficient of over 0.9 for the regression equation of this curve, which primarily indicates the curve’s stability. A higher correlation coefficient implies greater curve stability. Additionally, the data show a strong correlation between resistivity variation and deflection in intelligent HPC specimens across all fiber content levels.

According to the test results, the use of intelligent HPC mixed with fiber can effectively demonstrate the loading state in the bending state. The greatest changes in resistivity were observed when the carbon fiber content was 0.75% and the basalt content was 0.3%. These findings provide valuable data to support the implementation of self-monitoring in concrete structures.

### 3.4. Electrothermal Performance

The power-on heating performance is another important aspect that showcases the smart capabilities of HPC. This feature can be effectively utilized for melting ice and removing snow on roads and bridges located near structures in regions with seasonal freezing. By doing so, this significantly reduces the corrosion of structural components caused by deicing salts. Consequently, achieving the complete substitution of traditional snow removal methods necessitates HPC materials to meet stringent requirements for heating effectiveness.

As per the law of energy conservation, the continuous flow of a current against resistance results in the loss of energy, which is dissipated in various forms, such as thermal or mechanical energy. Generally, the energy lost by the resistance is expressed as thermal energy. When a voltage is applied to the resistance, electrons within it move towards areas with a higher electric potential due to the electric field. During this movement, electrons collide with molecules or atoms, producing heat energy. This microscopic phenomenon is also known as Joule heating.

By utilizing Joule’s law, this study aims to provide a reference for analyzing the temperature rise effect of concrete materials with varying electrical conductivity. This analysis will aid in selecting the optimal dosage and provide evidence for the feasibility of self-heating HPC structural materials removing snow.

To ensure a good heating effect, it is important to consider both the heating range and rate. In order to evaluate the heating effect of the test specimen, the heating range and rate were used as evaluation indices. Table 8 shows the heating status of the specific specimen during the electrothermal test.

**Figure 11:**
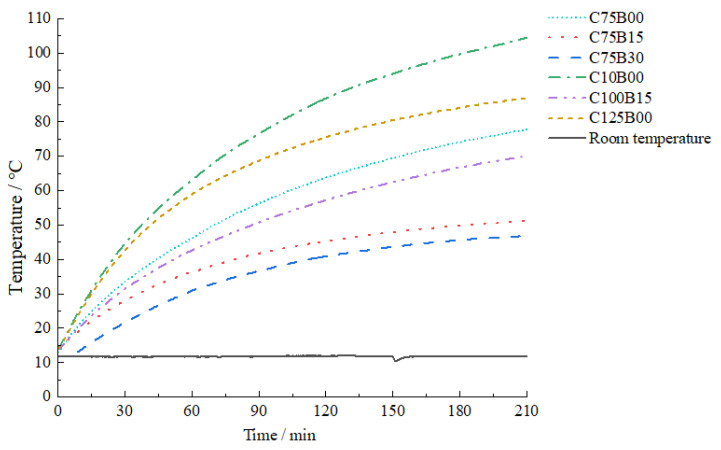
Heating effect of upper surface of intelligent hybrid fiber HPC in electrothermal test.

Figure 11 provides an in-depth analysis of the impacts and consequent trends of various fiber contents on the electrothermal heating effect of HPC.

The test results indicate that the intelligent HPC specimens with varying fiber contents exhibit a consistent increase in temperature, with a clear overall heating range. The temperature rises rapidly from the initial stage of electrification to 90 min, and gradually enters a gentler stage as the electrification time is extended. Each specimen demonstrates an excellent electrothermal performance.

The heating test results indicate a close relationship between the maximum temperature and heating rate of the specimen and the resistivity of the material with varying fiber contents. The maximum temperature varied significantly with different fiber contents, and the curve’s development trend also differed. The resistivity was at its lowest when the carbon fiber was doped at a content of only 1.0%, and the heating range was highest at 3.5 h. The heating curve was almost linear, with a noticeable heating effect on the specimen. The highest temperature on the upper surface reached 105.4 °C, and the heating rate was 25.86 °C/h. The C100B15 group demonstrated superior electrothermal performance when combined with carbon fiber, reaching a peak temperature of 70.9 °C and experiencing a rising rate of 16.31 °C/h over a period of 3.5 h. As the resistivity of the specimen decreased, the overall heating range increased, and the rate of temperature rise became more significant. According to Joule’s law, the smaller the resistance, the greater the heating power generated from the conversion of electric energy to thermal energy. Therefore, the electrothermal phenomenon becomes more apparent as more heat is generated. During electrothermal testing, it was observed that there was a difference in the heating rate and maximum temperature between the upper and lower surfaces of the specimen. This difference can be attributed to the fact that the electrode was not in complete contact with the lower surface during the pouring process, resulting in a gap. To reduce heat loss and resource waste, proper adjustment of the electrode position and distance is recommended during the arrangement of actual structural members.

The resistivity test results show that a carbon fiber content of up to 1.25% is consistent and does not result in significantly improved electrothermal properties. This is because the carbon fiber content has reached the percolation threshold range, completing the construction of a conductive fiber network, making it difficult to further reduce resistivity through the continuous addition of conductive fillers. When using carbon fiber alone, the optimum content should be 1% of the matrix volume. When mixed with basalt fiber, the optimum content should be 1% carbon fiber and 0.15% basalt fiber.

## 4. Conclusions

This study conducted a comprehensive analysis of the electrical properties of intelligent HPC, including resistivity tests and an analysis of the multi-scale pressure sensitivity, bending sensitivity, and electrothermal properties of intelligent HPC with varying fiber contents. The results provide evidence for the feasibility of using intelligent HPC materials in building structures. The study concludes with several key findings:

(1) The addition of carbon fiber to hybrid fiber HPC results in a significant decrease in resistivity, leading to excellent conductivity and providing a basis for the development of other intelligent functions. The resistivity of concrete generally increases with curing age, undergoing the three stages of rapid growth, slow rise, and stabilization. When the carbon fiber content reaches 1%, the percolation threshold is reached, resulting in the lowest resistivity. The addition of basalt fiber may decrease resistivity to a certain extent.
(2) The results of the pressure sensitivity test conducted on the specimens subjected to cyclic loading indicate that the resistance change rate follows a cyclical pattern, varying with the ratio of the fibers used. The addition of basalt fiber leads to an increase in pressure sensitivity. Among the groups tested, C75B30 exhibited the best varistor performance, with a maximum resistivity change rate of 41.62% and a sensitivity coefficient of 11.48.
(3) The results of the bending sensitivity test indicate that, while the addition of mixed fiber significantly improves the bending resistance of hybrid-fiber HPC, the brittle failure phenomenon is also obvious during failure. However, the HPC with different fiber contents showed good bending sensitivity. As the amount of basalt fiber increases, the maximum deflection and resistivity of the material also increase, indicating a positive hybrid-fiber effect. The material achieves maximum bending resistivity and sensitivity when the content of carbon fiber is 0.75% and the content of basalt is 0.3%.
(4) The hybrid-fiber HPC specimens with varying fiber contents exhibited a consistent increase in temperature, with a noticeable overall heating range. The temperature rapidly increased during the initial electrification and gradually transitioned into a gentler stage as the electrification time extended. Each specimen displayed exceptional electrothermal performance. At 3.5 h of electrification, the highest temperature recorded for the specimen was 104.5 °C, indicating the considerable potential for electrothermal ice-melting.

## Figures and Tables

**Table 1:** Physical properties and chemical composition.

	Ratio of Water Demand (%)	Water Content (%)	Ignition Loss (%)	MgO (%)	SO_3_ (%)	Cl^−^ (%)	SiO_2_ (%)
Cement	—	—	1.50	0.97	2.45	0.008	94.24
Silica fume	117	0.62	1.37	—	—	—	90.14
Fly ash	93	0.11	0.77	—	0.10	—	—
Granulated blast furnace slag powder	—	0.40	0.51	—	0.50	0.029	—

**Table 2:** The properties of the carbon fiber.

Carbon Fiber Type	Diameter (μm)	Length (mm)	Density (g/mm^3^)	Tensile Strength (MPa)	Carbon Content (%)
PAN-based	7	6	1.75	3500	95

**Table 3:** The properties of the basalt fiber.

Diameter (μm)	Length (mm)	Density (tex)	Breaking Strength (N/tex)	Breaking Elongation (%)	Moisture Content (%)
20	12	310–350	≥0.4	≥0.6	≤0.2

**Table 4:** Foundation mix ratio.

Water Binder Ratio	Cement (%)	Silica Fume (%)	Fly Ash (%)	Granulated Blast Furnace Slag Powder (%)	Sand Binder Ratio
0.18	62	13	5	20	0.7

**Table 5:** Fiber content.

	Serial Number	1	2	3	4	5	6	7	8	9	10
Fiber Type		C00 B00	C50 B00	C50 B15	C50 B30	C75 B00	C75B15	C75 B30	C100 B00	C100 B15	C125 B00
Carbon fiber content (%)		0	0.5	0.5	0.5	0.75	0.75	0.75	1.0	1.0	1.25
Basalt fiber content (%)		0	0	0.15	0.3	0	0.15	0.3	0	0.15	0

**Table 6:** Electrical property parameters of HPC materials with different fiber contents.

	Initial Resistivity (Ω·cm)	Peak Resistivity (Ω·cm)	FCR (%)	GF
C75B00	178.80	166.10	−7.10	1.63
C75B15	295.83	246.67	−16.62	4.02
C75B30	551.83	322.16	−41.62	11.48
C100B00	246.15	203.46	−17.34	4.18
C100B15	182.82	115.11	−37.04	8.17
C125B00	68.40	46.11	−32.59	7.93

**Table 7:** Flexural strength of intelligent HPC.

Specimen Number	Flexural Strength (Mpa)	Strength Increment (%)
C00B00	4.17	—
C75B00	7.78	86.55
C75B15	8.29	98.78
C75B30	8.54	104.68
C100B00	7.87	88.78
C100B15	8.67	107.99
C125B00	7.52	80.29

**Table 8:** Heating test status of each specimen.

NO.	Initial Temperature of Upper Surface/°C	Maximum Temperature of Upper Surface/°C	Upper Surface Heating Rate/°C·h^−1^
C75B00	13.1	78.0	18.54
C75B15	11.7	47.0	10.09
C75B30	14.2	51.7	10.71
C100B00	14	104.5	25.86
C100B15	13.8	70.9	16.31
C125B00	13.9	87.6	20.86

## Data Availability

Data are contained within the article.
